# Sliding Window-Based Region of Interest Extraction for Finger Vein Images

**DOI:** 10.3390/s130303799

**Published:** 2013-03-18

**Authors:** Lu Yang, Gongping Yang, Yilong Yin, Rongyang Xiao

**Affiliations:** School of Computer Science and Technology, Shandong University, Jinan 250101, China; E-Mails: yangluhi@163.com (L.Y.); ylyin@sdu.edu.cn (Y.Y.); canyueyang@126.com (R.X.)

**Keywords:** finger vein image, ROI extraction, sliding window, phalangeal joint, capture criteria

## Abstract

Region of Interest (ROI) extraction is a crucial step in an automatic finger vein recognition system. The aim of ROI extraction is to decide which part of the image is suitable for finger vein feature extraction. This paper proposes a finger vein ROI extraction method which is robust to finger displacement and rotation. First, we determine the middle line of the finger, which will be used to correct the image skew. Then, a sliding window is used to detect the phalangeal joints and further to ascertain the height of ROI. Last, for the corrective image with certain height, we will obtain the ROI by using the internal tangents of finger edges as the left and right boundary. The experimental results show that the proposed method can extract ROI more accurately and effectively compared with other methods, and thus improve the performance of finger vein identification system. Besides, to acquire the high quality finger vein image during the capture process, we propose eight criteria for finger vein capture from different aspects and these criteria should be helpful to some extent for finger vein capture.

## 1. Introduction

Biometric technology uses inherent behavior or physiological characteristics (e.g., fingerprints, faces, irises, finger vein, voices, gait, *etc.*) for personal identification with high security and convenience [1]. Compared with other biometrics, finger veins can be seen as a new biometric technology that is attracting more attention from the biometrics research community. Finger vein identification not only promises uniqueness and permanence like other biometric authentication techniques, but also has the following advantages over other biometric authentication techniques [1,2]: (1) contactless: non-invasive and non-contact data capture ensures cleanliness for the users, and can affectively avoid forging characteristics on the finger surface of users too; (2) live body identification: identification of finger vein patterns can only be taken on a live body; (3) high security: finger vein patterns are internal features that are difficult to forge.

Finger vein images are captured by near-infrared light (wavelengths between 700 and 1,000 nanometers) as shadow patterns, since near-infrared light can be absorbed intensively by the hemoglobin in the blood of vein, but easily transmitted by other finger tissues [3]. Many factors will influence the quality of finger vein images, for example, individual differences (*i.e.*, the fat thickness and skin color of different individuals are different), position of finger, image capturing environment and the performance of the image capturing device used [4]. Besides, no unified defined standard can be used to regulate image capturing, so it is inevitable that there are a certain number of low quality finger vein images in the captured images. We can classify low quality finger vein images into four types: (1) blurred image, there are less vein patterns with a low contrast in the images; (2) skewed image, when a finger in the finger vein image may show a certain degree of distortion; (3) very dark image, when there is a very dark part in the images; (4) very light image, with a very light part in the images. Low quality images will lead to unpromising identification results after time-consuming preprocessing and complicated feature extraction.

In the preprocessing of a finger vein identification system, the main task is localization and segmentation of the region of interest (ROI). ROI of a finger vein image refers to the region of finger which is filled with an abundant finger vein pattern network. The aim of ROI extraction is to decide which part of the image is fit for finger vein feature extraction, reserving the useful information in the ROI and removing the useless information in the background. The essential rule in extraction of ROI is that ROI should be available in all finger vein images from the database and there are sufficient finger vein features for extraction and comparison in the ROI. Accurate ROI extraction of a finger vein image will greatly reduce the computation complexity of any subsequent processing, and the most importantly, improve the performance of the finger vein identification system, so ROI extraction plays a critical role of finger vein identification systems.

Some finger vein ROI extraction algorithms have been proposed previously. Bakhtiar *et al.* [5] cropped the ROI using a fixed size window based on the center of the finger area in the finger vein image. This method is sensitive to finger displacement and is not available for skewed finger vein images. Yang *et al.* [6,7] proposed a ROI localization method based on the physiological structure of human fingers. Although this method can overcome the finger displacement issue, it was not suitable for skewed finger vein images either. If we want to use the existing ROI extraction methods, we must correct skewed finger vein images first. Kumar *et al.* [8] extracted ROI through an edge detector, and then performed rotational alignment, but this method did not crop the ROI area from finger vein images, and the preprocessed image has a large background region area.

Therefore, the limitations of the existing ROI extraction methods motivated us to explore an efficient and robust method for finger vein ROI extraction. This paper thus proposes a new ROI extraction method which can effectively resist finger displacement and rotation. In the method, we first detect the skew angle of the image and correct the image; then we obtain the height of the ROI based on the physiological structure of human fingers; lastly we make use of the internal tangents of finger's right and left edges to get the width of the ROI. In addition, through deep analysis of the finger vein capture process, and combined with our experience in finger vein capture, we propose eight capturing criteria to ensure high quality finger vein images.

The rest of this paper is organized as follows: Section 2 details the proposed finger vein ROI extraction method. Section 3 describes our experiments in detail, and discusses the experimental results. Section 4 presents some criteria for finger vein image capture. Finally, Section 5 concludes this paper.

## 2. Our Method

**Figure 1.**
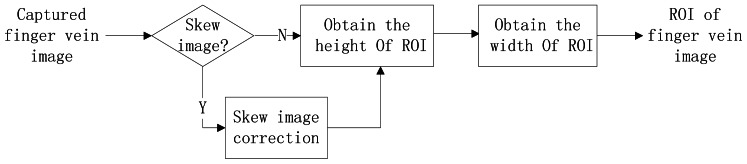
The block diagram of primary steps employed in ROI extraction.

In the captured image, there is a large background area with noise and useless information. Therefore the captured images are firstly subjected to ROI extraction for effectively and successfully carrying out the subsequent steps of the finger vein identification system. Figure 1 illustrates the block diagram of the primary steps employed in our ROI extraction method. There are three primary steps including: (1) detect and correct the skewed finger vein image, (2) determine the height of the ROI based on the phalangeal joints of the finger, (3) acquire the width of ROI based on internal tangents of finger's edges. The details of ROI extraction will be introduced in the rest of this section.

### 2.1. Skew Image Detection and Correction

Due to imperfect placement of fingers during image capture at different times, there is a certain amount of skewed finger vein images in which fingers show a certain degree of distortion. Therefore such images require skew correction. The correction of such distortion can assure that the proper expected area of each finger vein image can be extracted for accurate feature extraction and matching, and greatly improve the efficiency and correctness of the identification system. For all images in the database, we solve this problem in two substeps: (1) identify whether a finger vein image is skewed, and estimate the skew angle, (2) correct the skewed finger vein image based on the skew angle.

**Figure 2.**
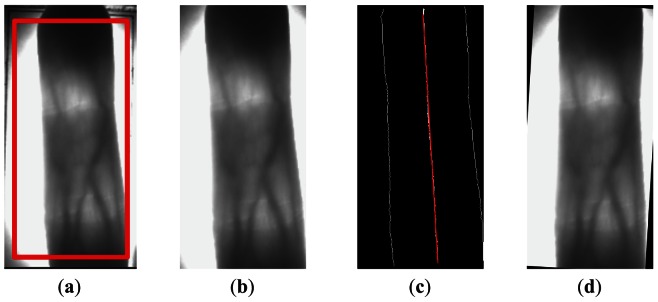
Skew image detection and correction. (**a**) A predefined window in red; (**b**) The finger vein candidate region; (**c**) The finger edges; (**d**) The corrected finger vein image.

We employ a linear fitting method to calculate the skew angle of a finger vein image. In detail, the discrete middle points of finger's right and left edges are synthesized into a straight line, and the angle between the synthesized straight line and the vertical direction is called as the skew angle of the finger vein image. The specific procedure of skew finger vein image detection is described in the following:

(1) Obtain ROI candidate region. A predefined window of 460 × 220 pixels in size is used to crop a finger vein candidate region for ROI extraction. For this process, we hold on two principles, including removing noises and useless information in background and reserving integrated finger region. The red window in Figure 2(a) is the predefined window and Figure 2(b) shows the finger vein candidate region.
(2) Detect the edges of the finger. The Sobel edge detector is applied to the finger vein candidate region and the resulting binary finger edge image is subtracted from the binarized image with denoising, shown as two white lines on left and right sides of Figure 2(c). This step is the most important in skewed image detection, because the edges of the finger are the foundation of our skewed image correction method. As we can see from Figure 2(c), the left edge of the finger is incomplete. The reason is, there is no different between the gray value of the finger region and that of the background in the top left corner of Figure 2(b), so the Sobel edge detector cannot detect the missing part of the edge, but we think it will not affect the estimation of the skew angle and the detected edges are enough for the skewed image detection.
(3) Figure out the midpoints of the finger edges. In a binary finger edge image, we figure out all rows, in which both edges of the finger can be detected, and in these rows the midpoints of the left and right edges of the finger are calculated. The midpoints is used to produce a straight line using a linear fitting method, and we call this straight line the middle line of the finger (shown as a white line in the middle of Figure 2(c)).
(4) Calculate and correct the skew angle. The difference value between the maximum column coordinate and the minimum column coordinate of all midpoints in per image is computed. If the difference value is less than 15 pixels, which is an empirical value, we think the image is normal, as the shape of the finger is an irregular rectangle. If the difference value is more than 15 pixels, the discrete middle points are synthesized into a straight line, and the skew angle of the finger vein image is obtained from the skew angle of the straight line.

In detail, (a) the equation of the straight line synthesized by all midpoints is shown in Equation (1), and the slope coefficient of the straight line is obtain, *i.e.*, a:

(1) y=a×x+b

(b) the tilt angle of the straight line, *i.e.*, α, is computed by Equation (2), and α is the skew angle of the finger vein image, which is used to correct the skew image:

(2) α=arctan(a)×360/2π

(c) the finger vein image is corrected based on the angle α, and the corrected image is shown in Figure 2(d).

### 2.2. Height Definition of ROI

**Figure 3.**
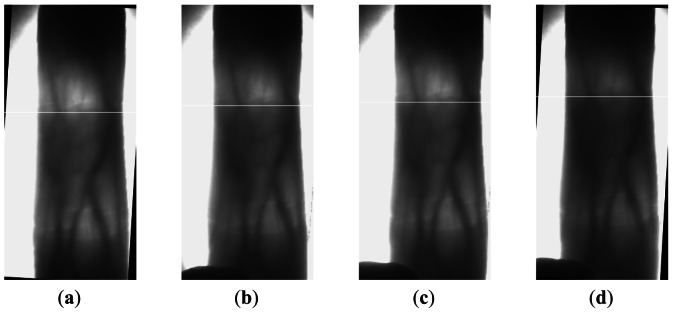
Locations of the distal inter-phalangeal joint in different images of one finger.

**Figure 4.**
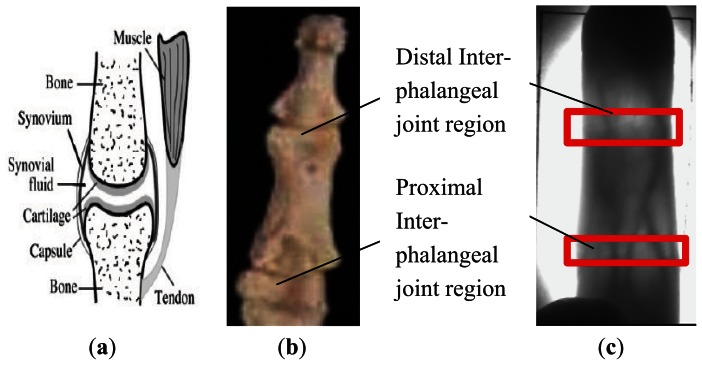
Phalangeal joint. (**a**) The structure of a phalangeal joint; (**b**) Phalanx structure; (**c**) Two phalangeal joint regions in finger vein image.

As fingers are displaced in the finger direction in finger vein image capture, the locations of the same section of a finger vary in different images of the finger. For example, the proximal inter-phalangeal joint of a finger is located in different positions in different images of the same finger, as seen in the white lines shown in Figure 3. In order to overcome this problem, we use two phalangeal joints, which are the distal inter-phalangeal joint and the proximal inter-phalangeal joint in the finger shown in Figure 4(b), as a reference to define the height of the ROI in different images of one finger.

A phalangeal joint consists of several components, including cartilage, articular cavity filled with synovial fluid, synovium and so on, as shown in Figure 4(a). The clearance between two cartilages allows more near-infrared light to penetrate, as the synovial fluid filling the clearance has lower density than that of bones. Thus two brighter regions, corresponding to two phalangeal joints, can be found in finger vein images, as shown in Figure 4(c).

**Figure 5.**
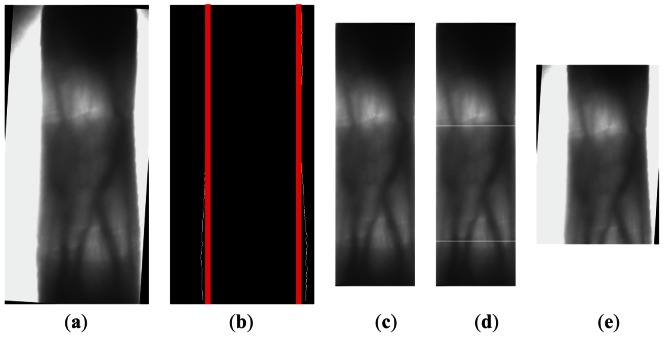
The height of ROI. (**a**) The corrective finger vein image. (**b**) The binary finger edge image with internal tangents. (**c**) The key area. (**d**) The detected positions of two phalangeal joints. (**e**)The ROI with defined height.

Through our deep observation of the finger vein image, we notice a phenomenon whereby the phalangeal joint may not always be located in the row with higher gray value of the finger vein image, so we make an improvement to the phalangeal joint detection method described in [6] with a sliding window, in which we will calculate the sum of gray values in the sliding window, not the sum of gray values in a row. The detailed procedure of achieving the height of ROI using a sliding window is as follows:

(1) Find the internal tangents of the finger's right and left edges. For the corrected finger vein image, the Sobel edge detector is applied again in the finger vein candidate region to get a binary finger edge image. For s normal finger vein image, it is not need to detect the finger edges again. In s binary finger edge image, we can get the internal tangents of finger edges, which are shown in Figure 5(b) in red. The key area can be obtained by cropping the region outside the internal tangents in finger vein candidate region, which will be used for detecting two phalangeal joints in next step. The key area is shown in Figure 5(c).
(2) Calculate the sum of gray value in a sliding window with 50 rows. We push the sliding window row by row from the top to the bottom of the key area. The sum of gray value is calculated using Equation (3). In Equation (3), w is the width of the key area:
  (3) $${\text{S}}_{\text{p}}=\sum_{\text{j}=1}^{\text{w}}\text{F}(\text{i},\text{j})\text{i}=1,2,\ldots ,50$$
(3) Estimate the position of two phalangeal joints. The position of phalangeal joint in the finger vein image generally has s higher row-sum value than the two side rows of a phalangeal joint, so the sliding window with the phalangeal joint will have the higher gray value. We separately use Equations (4) and (5) to determine the position of the distal inter-phalangeal joint and proximal inter-phalangeal joint.
  (4) $${\text{r}}_{1}=\text{arg}\;\text{max}\;({\text{S}}_{\text{p}})+49\;\text{p}\;\in \left[1,205\right]$$
  (5) $${\text{r}}_{2}=\text{arg}\;\text{max}({\text{S}}_{\text{p}})+({\text{r}}_{1}+100-1)+49\;\text{p}\;\in [{\text{r}}_{1}+100,411]$$
  where *r*_1_ denotes the position of the distal inter-phalangeal joint, *r*_2_ denotes the position of the proximal inter-phalangeal joint.

For the key area of the finger vein image, there will be 411 windows, so we search for the distal inter-phalangeal joint from the 1th window to the 205th window. We can establish a rule from the experiment results: the distance between two phalangeal joints is more than 100 rows, therefore we seek the proximal inter-phalangeal joint from the (r_1_ + 100) th window until the last window of the image. Besides, through careful observation of finger vein images, we find that the phalangeal joint is usually located in the bottom of the window, so the position of distal inter-phalangeal joint is the sum of the position of the window and 49. The white lines in Figure 5(d) denote the detected positions of two phalangeal joints:

(4) Define the height of the ROI. For one image, we need to calculate two distances:the d_1_ one is the distance between two phalangeal joints; the other d_2_ is the distance from the proximal inter-phalangeal joint to the last row of the image. Based on d_1_, d_2_, we utilize Equation (6) and Equation (7) to confirm the position of the ROI in height. For extracting more useful vein information, we expand the height of the ROI with d_1_×0.4 rows above r_1_ (*i.e.*, the distal inter-phalangeal joint) and d_1_ × 0.2 rows under r_2_ (*i.e.*, the proximal inter-phalangeal joint), so the distance between h_1_, h_2_ is the height of the ROI. Figure 5(e) shows ROI which is determined only in height, but not in width:
  (6) $${\text{h}}_{1}=\{\begin{array}{c} 1,{\text{r}}_{1}<{\text{d}}_{1}\times 0.4\\ {\text{r}}_{1}-{\text{d}}_{1}\times 0.4,\text{else} \end{array}$$
  (7) $${\text{h}}_{2}=\{\begin{array}{c} {\text{r}}_{2}+{\text{d}}_{1}\times 0.2,d_{2}<{\text{d}}_{1}\times 0.2\\ 460,\text{else} \end{array}$$

### 2.3. Width Definition of ROI

**Figure 6.**
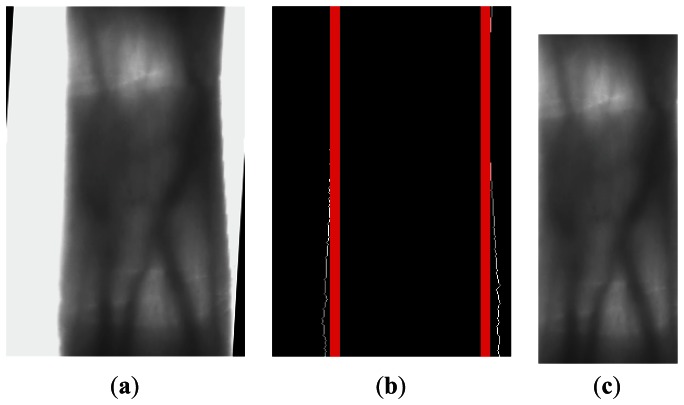
The width of the ROI. (**a**) The ROI with defined height. (**b**) The binary finger edge image with internal tangents. (**c**) The ROI of a finger vein image.

As the shape of the finger is an irregular rectangle, in different positions of the finger, the internal tangents of the finger edges are different. For a finger vein image, in which the height of the ROI is defined as shown in Figure 6(a), we need to detect the internal tangents of the finger edges again, as shown in red in Figure 6(b). The two internal tangents will be used as the left and right boundaries of the ROI, and we can obtain the ROI of a finger vein image, as shown in Figure 6(c). The purpose of width definition in this way is: (1) retain the maximum useful information in the finger region, (2) solve mismatching problems caused by finger displacement in the horizontal direction.

## 3. Experimental Results

### 3.1. The Experimental Database and Settings

**Figure 7.**
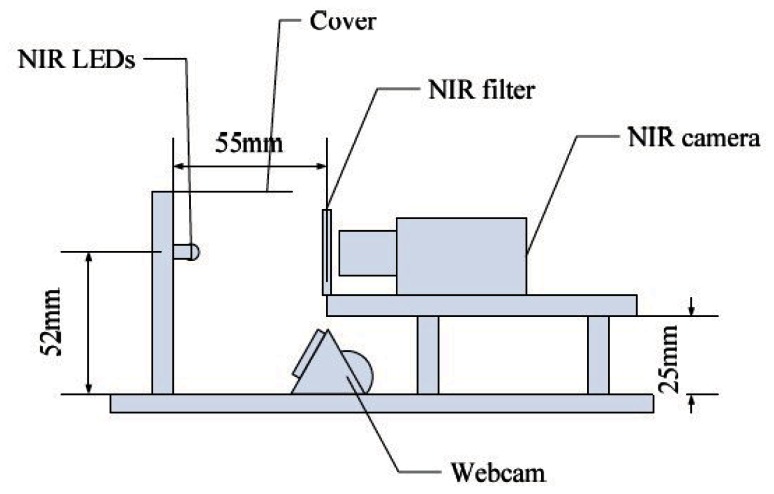
The imaging device.

In order to ascertain the performance of our ROI extraction method, we performed rigorous experiments on the finger vein image database from Hong Kong Polytechnic University [8]. The open database consists of 3,132 finger vein images taken from 156 volunteers over a period of eleven months using the imaging device shown in Figure 7. The dorsal side of finger is exposed to the near-infrared frontal surface illuminators, using light emitting diodes whose illumination peaks at 850 nm wavelength. The finger vein and finger texture images are respectively acquired by a NIR camera and a webcam (we only use the finger vein images captured by this device). As only 105 subjects turned up for the imaging during the second session, in our experiment, we use all finger vein images captured in first session including 1,872 finger vein images (156 subjects × 2 fingers × 6 images).

In order to prove the effectiveness of our proposed ROI extraction method, in this paper four experiments are designed to comprehensively evaluate the proposed method: (1) we compare our phalangeal joint detection method with the method in [6] in Experiment 1. (2) In Experiment 2, ROIs for skewed images with different skew angles are shown. (3) We extract the ROI for some typical finger vein images using different methods in Experiment 3. (4) Experiment 4 is designed to confirm our method leads to more accurate finger vein extraction performance compared with other ROI extraction methods.

### 3.2. Experiment 1

**Figure 8.**
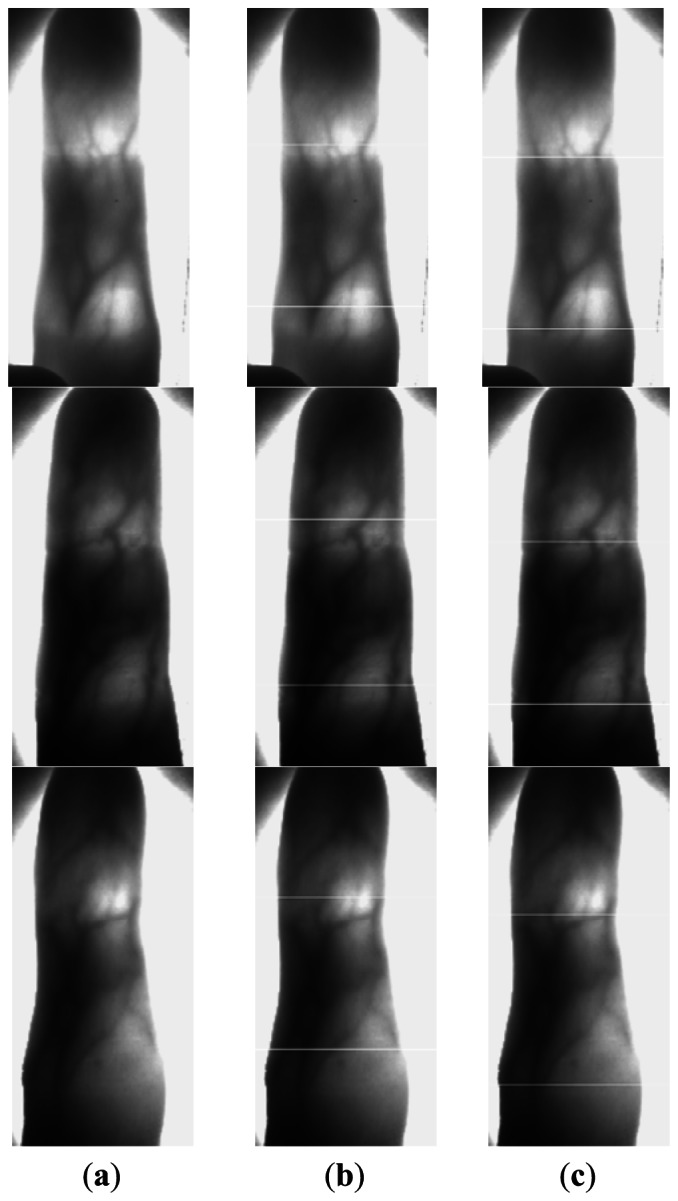
The positions of two phalangeal joints in the finger vein image. (**a**) The original finger vein image. (**b**) The positions of two phalangeal joints detected by the method in [6] (**c**) The positions of two phalangeal joints detected by our method.

In Section 2.2, we make an improvement to the phalangeal joint detection method in [6] with a sliding window. In this section, we compare our method and the method in [6] giving some images in Figure 8, in which the white line denotes the detected position of the phalangeal joint.

From Figure 8, we can see that our method works better than the other method. The phalangeal joint is not always located in the row with the higher gray value of the finger vein image, which may be affected by the uneven illumination. Therefore, we compute the sum of gray values in the sliding window with 50 rows, rather than compute the sum of gray values in a row.

### 3.3. Experiment 2

**Figure 9.**
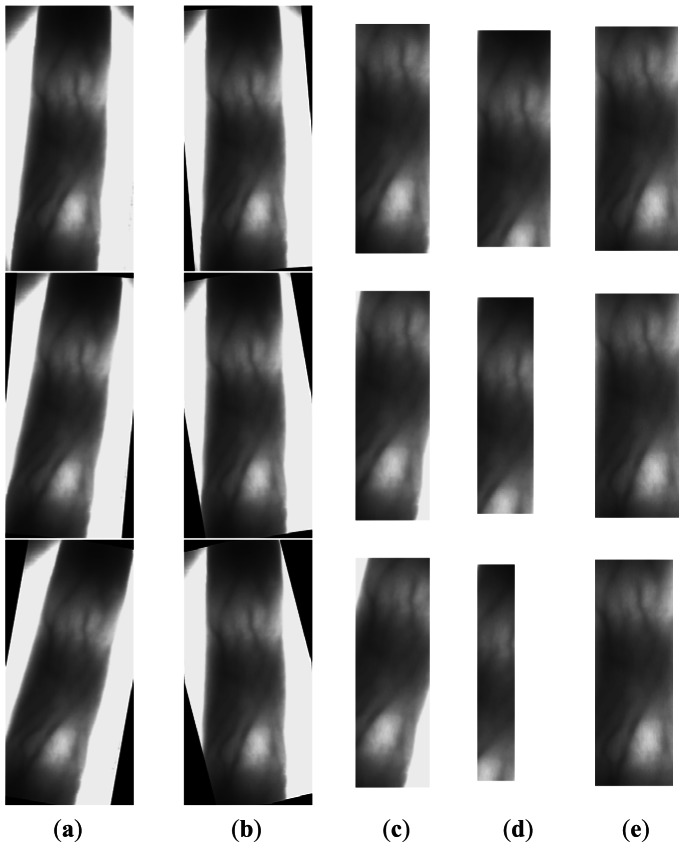
The effect of skewed image correction. (**a**) The skewed finger vein images. (**b**) The corrected finger vein images. (**c**) ROIs extracted by [5]. (**d**) ROIs extracted by [6]. (**e**) ROIs extracted by our method.

In this section, we first show the corrected finger vein images by our skew image correction method. We randomly select one skew finger vein image with a very small skew angle which has cut off noises and useless information around the captured finger vein images and only saved the finger vein candidate region. It is shown as the first figure in Figure 9(a). We artificially rotate the corrected image by − 5° and − 10° which are shown as the second and third figures in Figure 9(a), respectively. All the corrected images can be seen in Figure 9(b), and we can see, no matter how many degrees the image is rotated, our method can detect the skew angle and correct it effectively.

Next, we extract the ROIs of the images in Figure 9(a) using different methods. ROIs in Figure 9(c) extracted by a fixed size window have the same size, but the useless information in the ROI is added with increases the skew angle. Although the height of the ROIs in Figure 9(d) is constant, the width gets smaller and smaller. This is because the width depends on the internal tangents of the finger's edges, and the bigger the skew angle of the finger, the smaller the distance between the internal tangents. Our ROI extraction method works very well with the skew image detection and correction, from Figure 9(e).

### 3.4. Experiment 3

**Figure 10.**
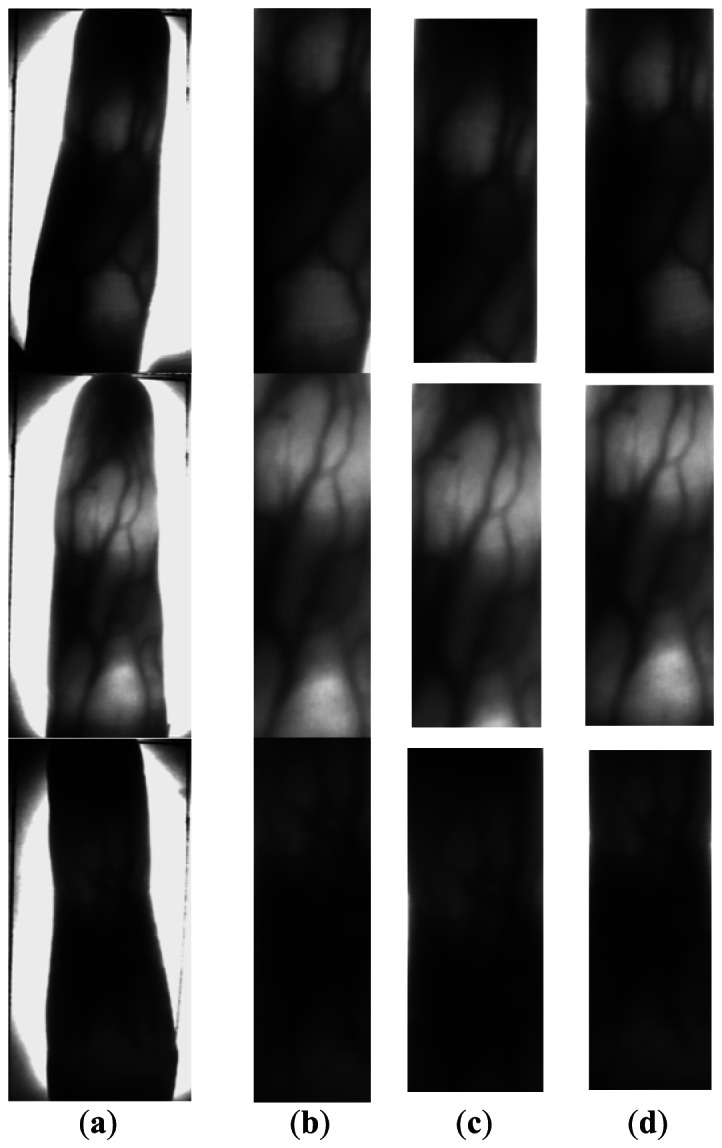
Some typical image samples and their ROIs. (**a**) Some typical finger vein images. (**b**) ROI extracted by [5]. (**c**) ROI extracted by [6]. (**d**) ROI extracted by our method.

Some typical finger vein images in our database and their ROIs extracted by different methods are shown in Figure 10. For Figure 10(b), the size of all ROIs extracted by a size fixed window is the same. As the size of the window must be available for all images, some images with big fingers will lose some useful information, for example, the second image in Figure 10(b). For Figure 10(c), the height of the ROIs is fixed, and lots of useful information is lost too. For Figure 10(d), although the size of the ROI is different, almost all the useful information is included in the ROI, and size normalization can be performed before finger vein feature extraction to solve the variable size problem.

We define the accuracy of the ROI extraction method as the area ratio between the automatically and manually segmented ROI. We randomly pick 100 images from the database to compute the accuracy of the above three ROI extraction methods. The average accuracies of 100 images are listed in Table 1.

The experimental results from Table 1 suggest that our method achieves the best performance among all the methods considered in this work. This may be a result of the skewed image detection and correction and use of phalangeal joints for ROI extraction. The poor performance of the methods in [5] and [6] may be limited by the two facts: (1) for skewed images, the width of the ROI, which depends on the internal tangents of finger edges, is too narrow; (2) The fixed width and height of the ROI will lose lots of useful information for some images.

### 3.5. Experiment 4

First the average time of our ROI extraction method is measured using 100 finger vein images randomly picked from the database. We implement our all experiments on a desktop computer equipped with 4 GB of RAM and an Intel Core i5-2400 CPU. The average ROI extraction time per image is 475 ms, which indicatess that our ROI extraction method can be used in real-time.

To further demonstrate that the proposed ROI extraction method has a major effect on the finger vein identification system, we designed Experiment 4. We compare the proposed method with other two methods used in [5] and [6], respectively. In finger vein identification, we perform size normalization to the ROI of finger vein images, so the size of the region used for vein feature extraction is reduced to 96 × 64 pixels. Besides, the local binary pattern (LBP) technique proposed in [9] is used for feature extraction and the hamming distance (HD) is used to estimate the similarities between the extracted binary codes and the enrolled code.

**Figure 11.**
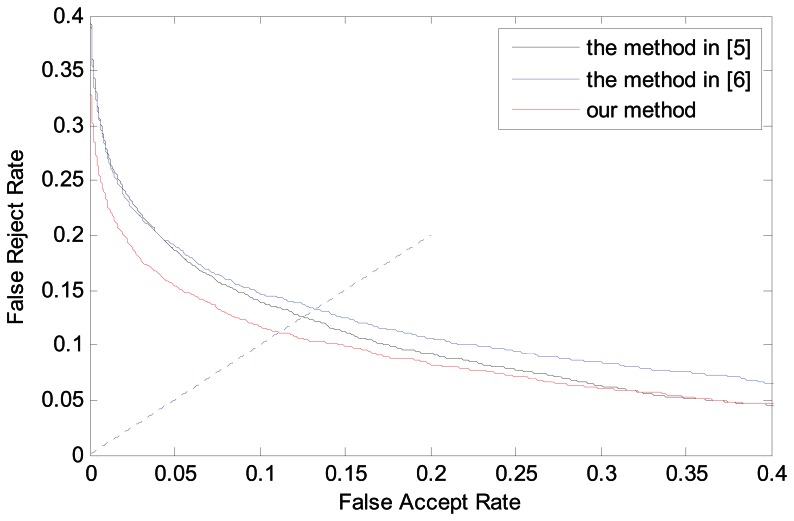
ROC curves by different ROI localization methods.

As described in Section 4.1, in our used finger vein image database, the finger vein class number is 312, and each class contains six finger vein images. Consequently, there are $4,680(312\times C_{6}^{2})$ intraclass matchings with six images in each class and $1,746,576(6\times 6\times {\text{C}}_{312}^{2})$ interclass matchings with six images in each class in total. The performance of the system is evaluated by the equal error rate (EER) as popularly used in benchmarking biometric systems. The experimental results from various approaches are summarized in Table 2, and the receiver operating characteristics (ROC) for the corresponding performances are illustrated in Figure 11.

It can be ascertained from Table 2 and Figure 11 that the proposed method achieves the best performance among all the approaches considered in this work. Specifically, the advantage of our method is mainly the result of the skewed image correction and the localization of two phalangeal joints in the image. As the size of fixed window in [5] must be appropriate for all images in the database, many images will lose lots of useful information, so the method in [5] has the results with highest EER value. Although the method in [6] can overcome the finger displacement, it does not work properly for skewed images.

**Figure 12.**
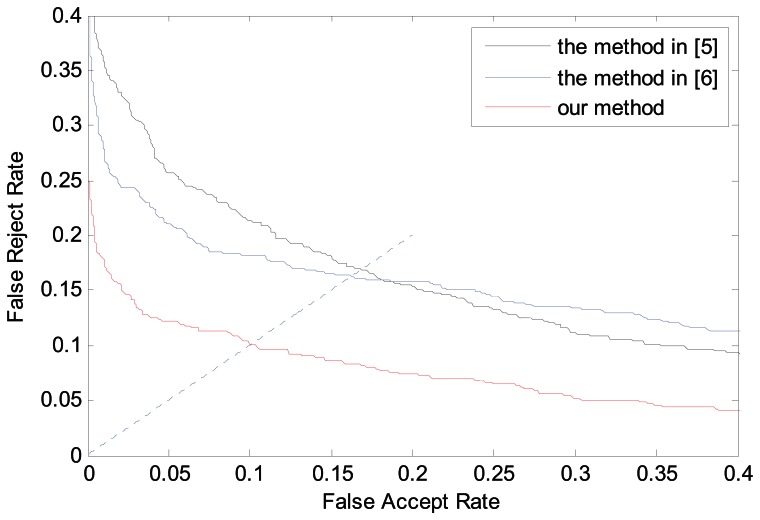
ROC curves on subdatabase by different ROI localization methods.

From the above experimental results, we can see that our method has no obvious advantage. The main reason is that the number of skewed images in the database is limited and most images are normal. In order to further show the advantage of our method in dealing with skewed images, finger vein images of 50 fingers are picked to perform the above experiment again, in which most images are skewed. The experimental settings are the same as above. The experimental results are shown in Table 3 and Figure 12, which can reflect the advantages of our method more apparently.

## 4. Criteria for Finger Vein Image Capture

In order to increase the usability and stability of finger vein identification systems, lots of works have been done [6,10–12], such as developing high-level finger vein recognition methods and employing robust venous enhancement methods, but one fundamental problem, which is neglected but directly related to the performance of finger vein identification system, is the quality of the captured finger vein images.

Influenced by many factors, there are a certain amount of low quality finger vein images. Fortunately, the quality evaluation of finger vein images has been researched [13]. Through the quality evaluation of images, we can filter out the low quality images or we can carry out special processing for low quality images, but this cannot fundamentally solve the problem of low quality finger vein images. In order to avoid low quality finger vein images, we must regulate the image capturing process and environment, and use a standard capture device. Therefore, to obtain high quality finger vein images, we propose some criteria for finger vein image capture:

(1) Suitable and stable illumination. The illumination intensity of the capture device has a significant impact on the quality of captured finger vein images. If the illumination intensity is very low, there will be lots of noise in the captured images, which will increase the difficulty of the follow-up processing. If the illumination intensity is very high, thin vein patterns will be lost. As we known, the near-infrared light with 700 nm–1,000 nm wavelength is best for capturing finger vein images, so near-infrared light with suitable intensity and light emitting diodes with stability performance are essential.
(2) Healthy and clean fingers. The finger of the subject which is used to capture finger vein images, should keep clean. Lots of factors will affect the penetration ability of near-infrared light, for example, moles, tumors and scars on the finger skin surface. When capturing finger vein images, we should choose healthy fingers without moles, tumors, scars or finger rings.
(3) The light transmission technique. There are two methods to grab the vein pattern: the light transmission technique and the light reflection technique [3]. In the light transmission technique, the dorsal side of the finger is exposed to the near infrared frontal surface illuminators, and the near-infrared light penetrates the finger and is captured by the device. However, in the light reflection technique, the image capture device and the light source are all on the front side of the finger, and the near-infrared light is reflected by the finger and captured by the device. Higher contrast finger vein patterns will be obtained using the light transmission technique, because the roughness and furrows on the skin's surface are obstructive to the detection of vein patterns under the light reflection technique.
(4) Correct finger position. The more finger vein patterns are extracted, the better identification performance will be achieved. When capturing images, fingers should be placed in the proper position, so that the distal inter-phalangeal joint, proximal inter-phalangeal joint and middle phalanx between the two joints are all in the captured finger vein image.
(5) Non-invasive and contactless data capture. As finger vein patterns are internal features and the near-infrared light penetrated the finger to capture finger vein imaged, the capture device should be designed to be user friendly and touchless. Non-invasive and contactless data capture ensures both convenience and cleanliness for the users, and it is more acceptable for the users.
(6) Feasible capturing environment. The captured finger vein image contains veins that have various widths and brightness, which may change with fluctuations in the amount of blood in vein, caused by changes in temperature [14,15]. For example, when we use finger veins for identification in very cold winter, the blood circulation in finger is not unblocked, so the identification mistakes can appear. The feasible capture temperature should be determined, avoiding image capture in very cold winter.
(7) Capturing images in different sessions. This mainly refers to the fact that the registered samples in the database should be captured in different sessions, and the interval between different sessions is two weeks at least or a longer time.
(8) Limited finger movement. In order to simulate the randomness of finger placement in image capture, when we capture the registered samples, we require the subject to rotate and move the captured finger. This action also can lead to negative effects: (a) if the position offset of the finger is very big, local vein information will be lost; (b) the random rotation and movement of finger may cause finger skew; (c) The phenomenon that the locations of the same section of a finger vary in different images of the finger, will appear. For (b) and (c), we can design a robust preprocessing algorithm to solve these issues, but for (a) the lost finger vein patterns are irretrievable. Therefore, it would be best to avoid big finger position offset and tilted fingerd in random finger movements.

In short, the high quality finger vein image is the basis of the good performance of finger vein identification system. Under these criteria, we can improve the quality of captured finger vein images, which is helpful for preprocessing, feature extraction and matching.

## 5. Conclusions and Future Work

In this paper, we present a robust ROI extraction method. We first detect and correct the image skew, then we make use of the phalangeal joints to ascertain the height of the ROI, and lastly we regard two internal tangents of the finger's edges as the left and right edge of the ROI. Two strengths of our method are the skewed image detection and correction and the definition of the ROI's height using a sliding window, which can overcome mismatching due to finger displacement. Compared with other ROI extraction methods, the proposed method works more accurately and effectively and leads to higher accurate performance, as shown from the experimental results. Besides, we present eight criteria for finger vein capture which will prove helpful to some extent for finger vein capture.

Through our deep observation of the finger vein image, we find a phenomenon that the phalangeal joint may not always located in the row with the higher gray value in the finger vein image. We guess the reason is the uneven illumination. In the future, we will explore the factors which cause this phenomenon.

## Figures and Tables

**Table 1.** The average accuracies of the different ROI extraction methods.

**Method**	**The Average Accuracies**
The method in [5]	0.8285
The method in [6]	0.8377
Our method	0.9415

**Table 2.** The performance of finger vein identification by different ROI localization methods.

**Method**	**EER**
The method in [5]	0.1256
The method in [6]	0.1330
Our method	0.1117

**Table 3.** The performance of finger vein identification on subdatabase by different ROI localization methods.

**Method**	**EER**
The method in [5]	0.1680
The method in [6]	0.1625
Our method	0.1015
